# Case-specific potentiation of glioblastoma drugs by pterostilbene

**DOI:** 10.18632/oncotarget.12298

**Published:** 2016-09-28

**Authors:** Linnéa Schmidt, Sathishkumar Baskaran, Patrik Johansson, Narendra Padhan, Damian Matuszewski, Lydia C Green, Ludmila Elfineh, Shimei Wee, Maria Häggblad, Ulf Martens, Bengt Westermark, Karin Forsberg-Nilsson, Lene Uhrbom, Lena Claesson-Welsh, Michael Andäng, Ida-Maria Sintorn, Bo Lundgren, Ingrid Lönnstedt, Cecilia Krona, Sven Nelander

**Affiliations:** ^1^ Life Laboratory, Uppsala University, Uppsala Sweden; ^2^ Centre for Image Analysis, Department of Information Technology, Uppsala University, Uppsala, Sweden; ^3^ Sahlgrenska Cancer Center, Institute of Medicine, Gothenburg, Sweden; ^4^ Department of Physiology and Pharmacology, Karolinska Institute, Sweden; ^5^ Cell Screening Facility, Science for Life Laboratory Stockholm, Department of Biochemistry and Biophysics, Stockholm University, Solna, Sweden

**Keywords:** glioblastoma, glioblastoma initiating cells, stilbenoids, drug repurposing, cancer therapeutics

## Abstract

Glioblastoma multiforme (GBM, astrocytoma grade IV) is the most common malignant primary brain tumor in adults. Addressing the shortage of effective treatment options for this cancer, we explored repurposing of existing drugs into combinations with potent activity against GBM cells. We report that the phytoalexin pterostilbene is a potentiator of two drugs with previously reported anti-GBM activity, the EGFR inhibitor gefitinib and the antidepressant sertraline. Combinations of either of these two compounds with pterostilbene suppress cell growth, viability, sphere formation and inhibit migration in tumor GBM cell (GC) cultures. The potentiating effect of pterostilbene was observed to a varying degree across a panel of 41 patient-derived GCs, and correlated in a case specific manner with the presence of missense mutation of EGFR and PIK3CA and a focal deletion of the chromosomal region 1p32. We identify pterostilbene-induced cell cycle arrest, synergistic inhibition of MAPK activity and induction of Thioredoxin interacting protein (TXNIP) as possible mechanisms behind pterostilbene's effect. Our results highlight a nontoxic stilbenoid compound as a modulator of anticancer drug response, and indicate that pterostilbene might be used to modulate two anticancer compounds in well-defined sets of GBM patients.

## INTRODUCTION

The dismal outcome for glioblastoma (GBM) patients with current therapies [1] strongly motivates the exploration for new therapeutic approaches. Amounting evidence suggests that GBM cells can be inhibited by synergistically acting pairs of compounds [2–5], such as tricyclic antidepressants together with inhibitors of the P2Y_12_ receptor family of purinergic G protein coupled receptors, or antidepressants with sigma receptor inhibitors [4, 6]. Such repurposed combinations of approved drugs offer a faster route to clinical evaluation and it is therefore a priority objective to determine which pathways are relevant for combinatorial targeting of GBM. Furthermore, the fact that GBM tumors exhibit molecular heterogeneity, prompts the questions to what degree transcriptional subtypes of GBM [7] might affect drug-drug synergy, and if there are safe compounds that can significantly potentiate existing drugs in well-defined subsets of GBM patients.

Pterostilbene (trans-3, 5-dimethoxy-4′-hydroxystilbene) is chemically classified as a stilbenoid and biologically as a phytoalexin (a class of low molecular weight compounds synthesized by plants as part of their antimicrobial defense). It is considered a safe compound with no reported toxicities, and is found naturally in berries [8–13]. Analyses of both pterostilbene and its analog resveratrol (3, 5, 4′-trihydroxy-trans-stilbene) have indicated anticancer effects in cell-based and mouse experimental systems [14]. Evaluation in rodent cancer models has shown that pterostilbene can suppress the progression of experimental colon and liver cancers [15]. In a model of colon cancer, pterostilbene suppressed beta-catenin and cyclin D1 in colon cancer tumor samples [16]. The molecular basis mechanism of stilbenoids is not fully understood; but phosphodiesterase (PDE) enzymes [17], cyclooxygenase and in particular cytochrome p450 components have been implicated as possible targets [18]. Studies of pterostilbene in brain cancers are limited to the previous study from our lab [4] and one recent publication showing that pterostilbene suppresses self-renewal, irradiation-resistance and gliomagenesis *in vivo* [19], however it has shown activity in model systems of other cancer types [16, 20, 21]. Pterostilbene is also relevant for glioma treatment due to its high bioavailability and its ability to pass the blood brain barrier [8, 11].

A recent large scale screen detected that pterostilbene might functionally interact with other compounds to suppress growth in GBM [4]. Two such tentative interacting partners were the serotonin reuptake inhibitor (SSRI) sertraline and the EGFR tyrosine kinase inhibitor gefitinib. Sertraline, while not intended as a cancer drug, effectively passes the blood brain barrier; it has been reported to have activity against GBM cells [7, 22], and is being considered for clinical evaluation in GBM patients [23]. The target of gefitinib, EGFR, is frequently altered in GBM, by point mutation, chromosomal aberration, or both [24, 25]. However, clinical trials of gefitinib have not shown a significant increase in GBM patient survival [26]. It is therefore interesting to consider pterostilbene as a possible modulator of clinical response to both sertraline and gefitinib.

We analyzed the effect of pterostilbene as a potentiating compound across a panel of glioblastoma cell (GC) cultures [7, 27, 28] established from patient surgical samples. By sampling GCs from several patients, we could assess variations in the level of functional interaction between pterostilbene, gefitinib and sertraline across a large and diverse sample of patient-derived cell cultures. Further, we explored how pterostilbene, singly or in combination, suppressed malignant phenotypes in GCs, such as migration and proliferation, and investigated the mechanism by which pterostilbene modulates sertraline and gefitinib. The results identify pterostilbene as a potentiator of two drugs with anti-GBM activity with possible implications for other malignancies.

## RESULTS

### Pterostilbene potentiates gefitinib and sertraline to suppress malignant phenotypes of GCs

**Figure 1:**
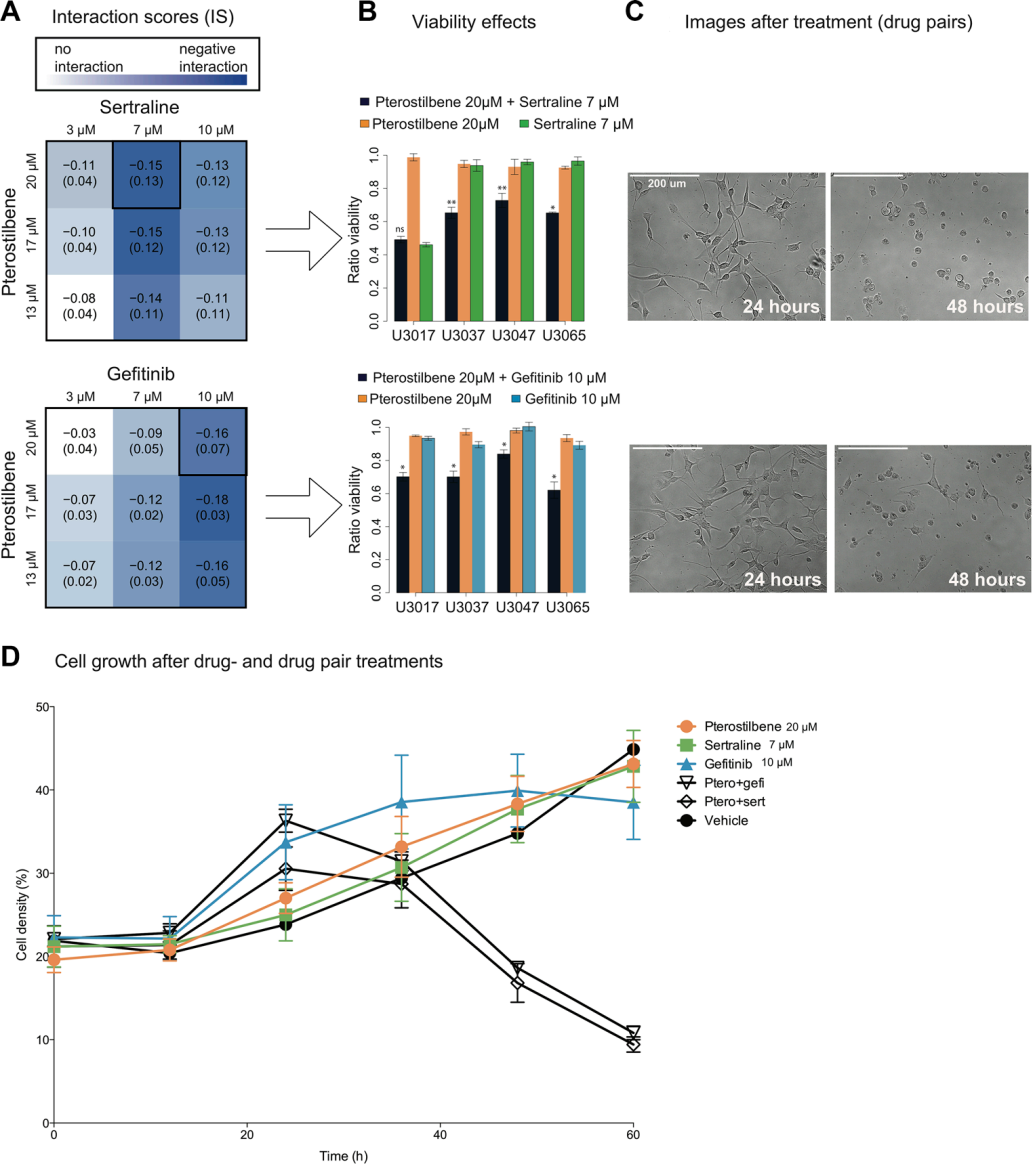
Combination of pterostilbene with sertraline or gefitinib suppresses glioma cell growth 72 hours viability response to sertraline, gefitinib and pterostilbene, in four GCs treated in triplicates for each dose and combination. (**A**) Evaluating each compound at three different doses, we obtained pairwise interaction scores (IS, Methods). A negative IS, consistent with a potentiating interaction, was more pronounced at particular dose concentrations. Numbers in the table are mean and SD of IS across GCs (*n* = 4). (**B**) Combinations of pterostilbene with sertraline or gefitinib suppressed viability at 72 hours, whereas the single agents did not. (error bars are 95% CI). Both combinations display a significant interaction score at the doses tested in all GCs except for the PS combination in U3017. *p < 0.05, **p < 0.01 (Methods). (**C**) Images of treated GC U3065 at 24 and 48 hours. (**D**) Real time cell growth density measurements recorded every 12 hours for 60 hours in U3065, presented as cell growth curves with mean and SD from triplicates for each recorded time point.

We first investigated the effect of pterostilbene, gefitinib and sertraline (Supplementary Figure S1A) in a set of four glioblastoma cell (GC) cultures (U3017MG, U3037MG, U3047MG and U3065MG). In each of the cultures, we measured the viability following treatment by pterostilbene, sertraline and gefitinib, applied singly and in combination. The responses were used to calculate an *Interaction Score* (IS, Methods). A negative IS (IS < 0, indicating an interaction of a potentiating type) was observed between pterostilbene and each of gefitinib and sertraline, at multiple dose combinations (Figure 1A). As a working model for downstream experiments, we chose a set of doses that consistently gave a negative score in all four GC cultures (20 μM pterostilbene, 7 μM sertraline and 10 μM gefitinib, Figure 1B). For these doses, the pterostilbene + gefitinib (PG) and pterostilbene + sertraline (PS) pairs significantly suppressed cell viability whereas single compounds did not (IS < 0, Figure 1B–1C). Additional analysis of the time dependency of the response showed that PS and PG negative interaction (IS < 0) becomes apparent after approximately 35 hours of combination treatment (Figure 1D).

**Figure 2:**
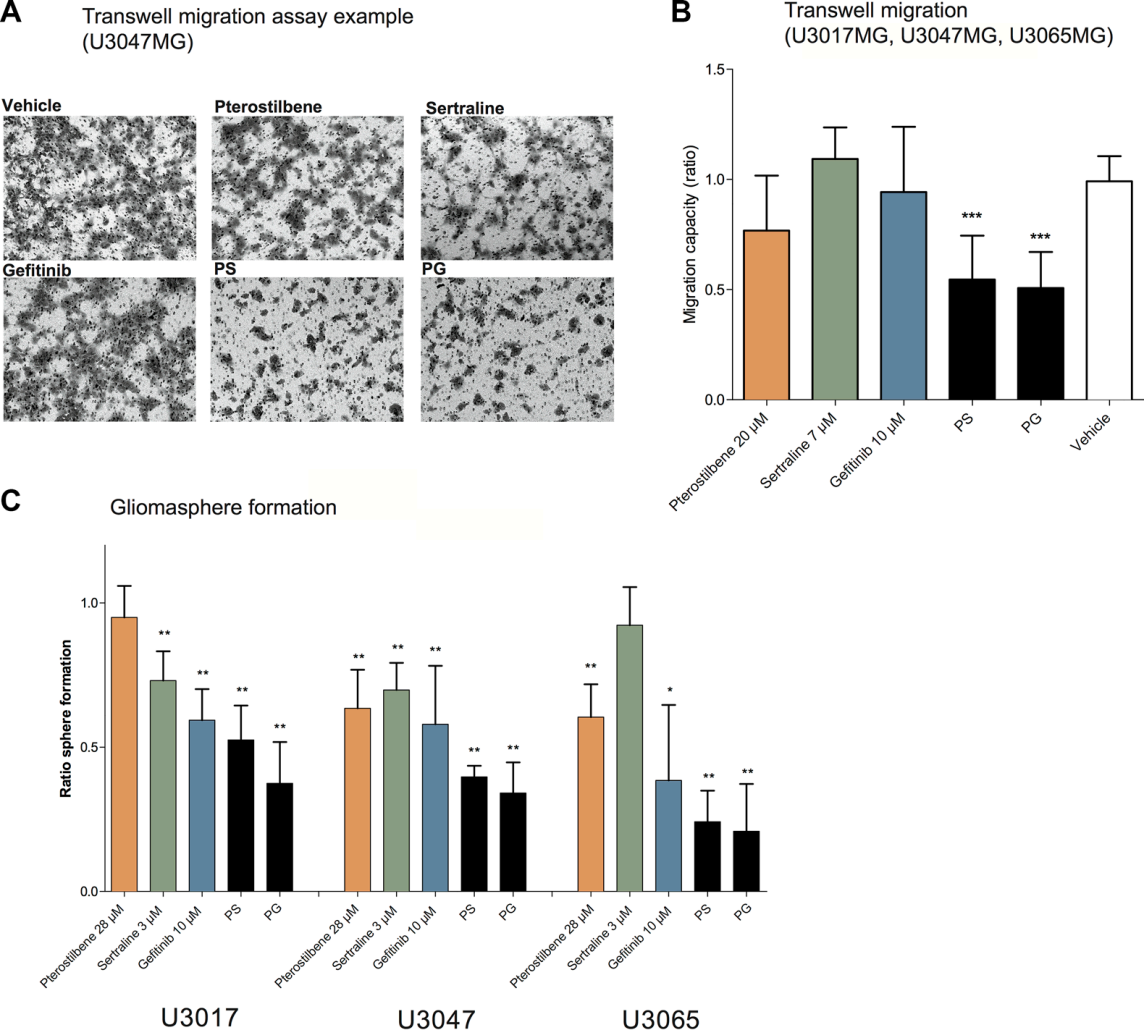
Combination of pterostilbene with sertraline or gefitinib affect glioma cell migration and sphere formation (**A**) Images of migration trans-well membranes after treatment (U3047). (**B**) Trans-well migration capacity after 48 hours of treatment in GC cultures. Graphs are plotted from the collected results from experiments in three GC cultures (U3047, U3065 and U3017, a total of 9 ratio data points for each treatment). (**C**) Gliomasphere formation after 7 days of treatment (experiment in six replicates for each treatment in U3047, U3065 and U3037). All ratios are calculated from vehicle control measurements, and all graphs are presented as means with 95% confidence interval. *p < 0.05, **p < 0.01, ***p < 0.001 (Mann Whitney).

In addition to a synergistic effect on cell viability, the PS and PG pairs also suppressed cell migration and gliomasphere formation in the GC cultures (Figure 2). Thus, while the single drugs displayed a modest effect on migration in the GCs tested, the PS and PG pairs significantly suppressed migration in U3017MG, U3047MG and U3065MG (*p* < 0.05) (Figure 2A, 2B). Furthermore, both PS and PG combinations displayed a significant inhibitory effect on gliomasphere formation (Figure 2C and Supplementary Figure S1B) in U3017MG, U3047MG and U3065MG (*p* < 0.05). For the migration and clone formation assays, U3017MG and U3037MG were challenging cultures to work with. As a result of this, U3037MG was excluded from the gliomasphere forming- and migration analysis and U3017MG from the EdU proliferation assay.

Altogether, the PS and PG pairs were demonstrated to suppress viability, migration, and sphere forming capacity of GC cultures.

### Investigating drug interactions in cells from 41 different patients

Next, we asked if PS and PG synergy would be consistently observed across a larger sample of GCs cultures from different individuals. We thus measured the response to PS and PG across an extended set of 41 patient-derived GC cultures from our Human Glioma Cell Culture (HGCC) biobank [29]. The cultures were obtained from 25 males and 16 females (average age of 65.5 years) assigned a pathological diagnosis of WHO astrocytoma grade IV, or glioblastoma multiforme (GBM). Among the 41 cell cultures, all molecular subclasses proposed for GBM are represented (Supplementary Table S1) [25] and all cells used were IDH1 wildtype as determined by exome sequencing [29].

**Figure 3:**
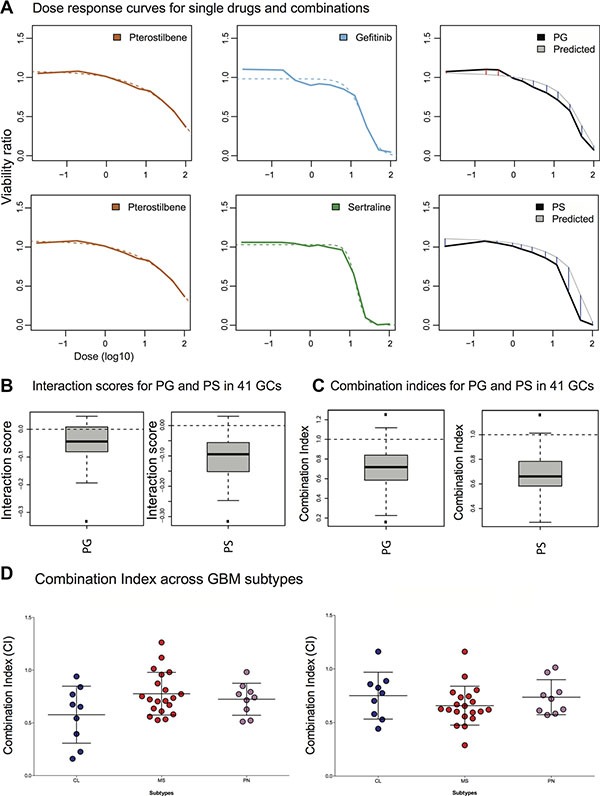
Measurement of pterostilbene induced potentiation across 41 GC cultures A screening experiment was performed, measuring 11 dose series of each of the drugs both single and as fixed-proportion concentrations for the combinations pterostilbene and gefitinib (PG) and pterostilbene and sertraline (PS) in 41 GCs. (**A**) Sigmoid dose response curves (dashed lines) were fitted to the mean viability ratio across the 41 GCs (orange, blue and green lines and results for individual GCs in Supplementary Figure S2). Each two sigmoid curves for single drugs (left and middle panel) were used to derive an expected combination response curve under the assumption of no synergy (grey line). The thick black lines (right panel) show the average observed combination response (doses in log10). Blue and red lines represent a synergy or an antagonism, respectively. (**B**, **C**) Boxplots of the patientwise average IS (across all overlapping doses tested) and patientwise average CI (effect 40–60%) across all 41 GCs. The results confirm a significant generality of the synergistic response for the drug pairs in a large patient material (*p* < 0.0001, Methods). (**D**) Differences in CI across the GC panel annotated by their subtype. CI for PG and PS, left and right, respectively. No statistical difference was obtained between subtypes (ANOVA *p* = 0.075, main text), although a trend towards a stronger synergy for the PG combination in the Classical subtype was observed.

Using an innovative protocol, we evaluated each drug at 11 individual doses and further evaluated the PS and PG pairs at 11 different doses in a fixed ratio (Supplementary Table S2), after 72 hours of drug exposure. From these three dose series, we estimated both the interaction score (IS) and the *Combination Index* (CI, Methods) (Figure 3A–3C and Supplementary Figure S2A–S2C). Across the 41 cases, the mean IS of pterostilbene+sertraline (PS) and pterostilbene+gefitinib (PG) were negative, −0.10 and −0.05 respectively, indicating a functional interaction consistent with synergism. Moreover, the mean CI was below 1, 0.65 and 0.7 for PS and PG respectively, demonstrating pairwise synergism (Figure 3B, 3C). Both IS and CI medians were significantly different from 0 and 1, respectively (IS and CI for PG and PS *p* < 0.0001). As a point of reference, human astrocyte cultures were comparatively less sensitive to pterostilbene (Supplementary Figure S3).

Taken together, the initial observation that pterostilbene potentiates gefitinib and sertraline in GBM was thus extended to a broad set of patient-derived samples and shown for two metrics of functional interaction.

### Pterostilbene induced drug potentiation can be predicted in individual GC cultures

We next asked if pterostilbene-induced potentiation of gefitinib and sertraline would correlate with either clinical variables or molecular signatures. For the analyses we focused on CI, rather than IS, since CI is the metric that is often used to formally assign ‘synergism’ relationships between drug pairs and since the estimation of CI involves more data points and is therefore more robust (Methods). Whereas no correlation was observed between CI and age, gender or survival (days from diagnosis to death) for the PG combination, the CI of the PS pair correlated with age (*p* < 0.05, Supplementary Figure S4) (The application of correlation to survival times is adequate since all patients were diseased, i.e. no censored data points). We proceeded to test for differences in CI across each of the GBM molecular subtypes GBM [25]; Classical (CL), Mesenchymal (MS), Neural (NL) and Proneural (PN), assigned as in [30]. Since only two of the GCs were annotated for the NL subtype, the analysis was focused to the three main subtypes. The PG pair tended towards elevated synergism (lower CI) for the CL subtype (ANOVA *p* = 0.075 for a difference between the three groups, Student's *t*-test *p* < 0.05 compared to the MS subtype, and *p* = 0.17 when compared to the PN subtype), but the ANOVA does not support a significant difference between the groups collectively (Figure 3D).

**Figure 4:**
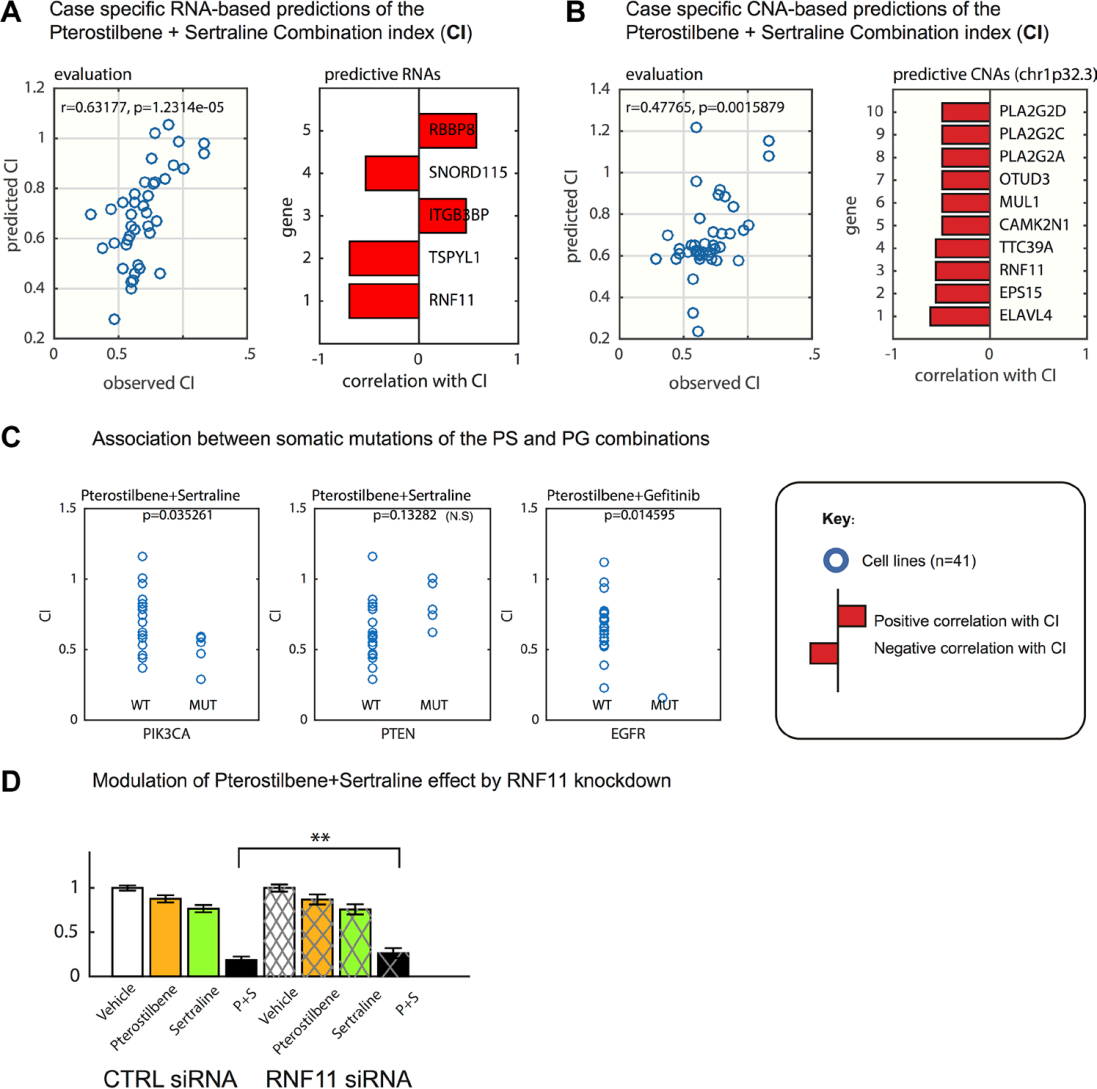
Predictive markers of synergy between pterostilbene, sertraline and gefitinib in glioblastoma cell (GC) cultures Using data from 41 GC cultures, we applied elastic net regression to predict the Combination Index (CI) between pterostilbene and sertraline. (**A**) Prediction of PS CI could be predicted with good accuracy from RNA transcripts (Pearson correlation of 0.63 between X = observed and Y = predicted values as obtained by cross-validation, left). Transcripts selected as predictive transcripts (showing coefficients as bars) included RNF11 and TSPYL1 (right). (**B**) The same analysis performed using DNA copy number aberration data for the GC cultures. The predictive performance is not as high as for transcripts (left), and the model identifies a set of genes encoded by the same region on chromosome 1p (including the RNF11 locus) as predictive. (**C**) Association between PS CI and PIK3CA missense somatic mutation status (left) and PG CI and EGFR status (right). PIK3CA missense somatic mutation status (left), PS CI and PTEN missense mutation status (middle) and PG CI and EGFR status (right). (**D**) Modulation of PS combination effects during simultaneous knockdown of RNF11 (24 and 48 h experiments collected) (*p*-value obtained by linear model, Methods)

Using a complementary method to associate CI with molecular profiles, we applied elastic net regression with variable selection [31] to detect molecular changes that would predict variations in CI. The analysis showed that both transcripts and DNA copy number aberrations in the GC cultures could predict CI for the PS pair, as shown by a leave-one-out cross validation analysis (Figure 4). The procedure selected a total of five transcripts as optimally predictive of PS synergy. The transcript that correlated most with PS Combination Index was Ring Finger 11 (RNF11), a component of a ubiquitin editing complex with broad functions, including modulation of cellular internalization of the EGF receptor [32] (Figure 4A). Interestingly, the elastic net procedure, when applied to DNA copy number data instead of expression data as the predictor of CI, identified the chromosomal locus of RNF11 (chr1p32.3) as predictive. This locus contains not only RNF11 but also a set of phospholipases and other genes (Figure 4B). To explore the functional relevance of this association, we suppressed expression of RNF11 by siRNA knockdown in a cell line with normal DNA copy number of the RNF11 locus, U3027MG. RNF11 knockdown protected the U3027MG cells from the PS combination to a moderate but significant degree (*p* < 0.01) (Figure 4D). This finding suggests a possible role for RNF11 in modulating the response to the PS pair. For the PG pair, application of the elastic net method did not result in a gain in predictive performance over transcriptional signatures (not shown).

Finally, we analyzed if the CI for PS and PG, respectively, was correlated to missense somatic mutation of protein coding genes in the GC cultures (unpublished data). We performed an analysis in which somatic mutations in the 10 genes with numerically highest somatic mutation frequency in the hgcc.se biobank (EGFR, NF1, PDGFRA, PIK3CA, PIK3R1, PTEN, RB1, RYR2, TP53, TTN) each were analyzed as possible predictors of Combination Index. For the PG combination, CI was significantly lower in the one cell culture with a high stringency (Methods) mutation in the EGFR gene (*z* test *p* = 0.015). The low frequency of EGFR mutant cell cultures is partly a sampling coincidence (the 41 cell cultures are part of a material with 9% EGFR detected missense point mutation frequency) but may also reflect the stringent use of mutation callers by the hgcc.se consortium (Methods). No IDH1 mutation was found among our cell cultures. The CI of the PS combination, in turn, was statistically associated with PIK3CA somatic missense mutation (*t* test *p* = 0.035) and showed borderline significance with PTEN somatic missense mutations (*p* = 0.13) (Figure 4C).

Taken together, while further analysis of additional EGFR mutant cell cultures is warranted, our observation of lower CI in classical subtype glioma and one EGFR mutant line appears consistent with the hypothesis that the PG pair is more synergistic in GC lines with a classical GBM signature with hyperactivated EGFR signaling. However, due to the low numbers of mutated samples these findings would benefit from being confirmed in larger studies. For PS, we identify RNF11 transcripts and the RNF11-encoding chromosome segment on Chr1p32 as possible biomarkers of pterostilbene mediated potentiation. In future work, it may be possible to extend on these findings to stratify GBM into cases more likely to benefit from pterostilbene containing drug combinations.

### Pterostilbene potentiates gefitinib and sertraline in GCs by cell cycle arrest and suppression of the MAPK pathway

**Figure 5:**
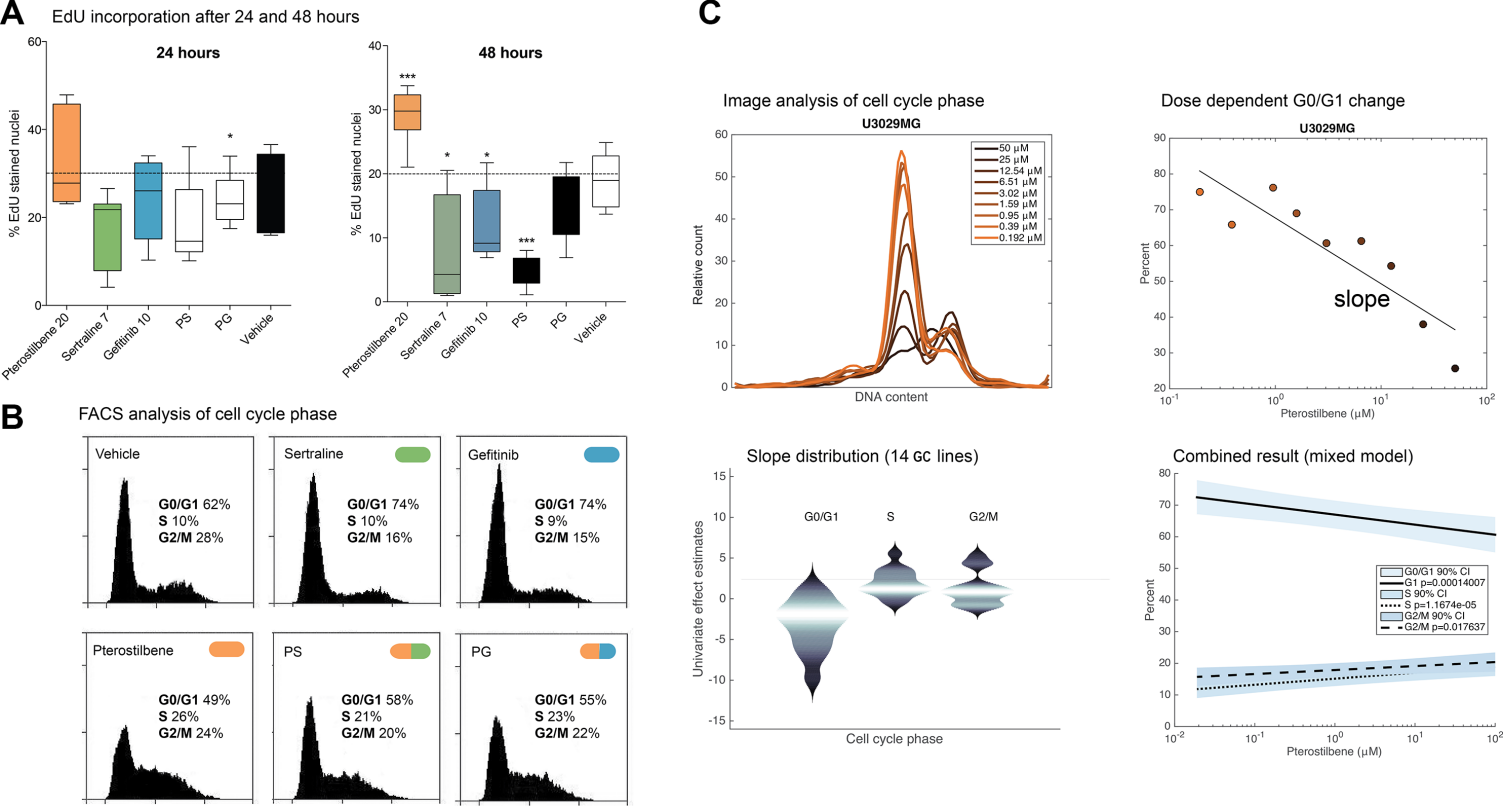
Drug combinations and single drugs affect proliferation, and pterostilbene modulates cell cycle progression Assessment of proliferation and cell cycle analysis after treatment with single drugs and combinations. (**A**) EdU incorporation (% positive cells) after 24 and 48 hours. Box and whiskers represent mean and 5–95 percentiles from experiments in three GC cultures (all data plotted in Figure S5B–S5C). *p < 0.05, **p < 0.01, ***p < 0.001 (Mann Whitney) (**B**) Flow cytometry of 7AAD-stained cells (U3065) to measure the distribution of DNA content in treated cells. An accumulation of cells in the center of the DNA content distribution compared to control sample is indicative of cell cycle arrest with an enriched S-phase population. The pterostilbene and combinations display an evident cell cycle shift in this GC culture. (**C**) Automated imaging of GC nuclei was used to derive histograms of cell DNA content under treatment with different doses of pterostilbene, upper left. Upper right: to quantify, we fitted GC specific linear regressions in which each of the G0/G1, S and G2/M populations were proportional to the logarithmic dose of pterostilbene (G0/G1 in one GC culture shown). The graph (lower left) shows the distribution of the proportionality (slope) constants across the 14 lines for each population. Lower right: linear mixed effects model result, showing the estimate and 95% confidence interval of a common (‘fixed’) slope effect across the 14 cell cultures (*p*-values 0.00014, 1.17 × 10^−05^ and 0.018 for the G1, S and G2/M populations, respectively).

Since pterostilbene was previously reported to induce cell cycle arrest in leukemia and carcinoma cells [21, 33, 34], we investigated the effect of pterostilbene and PS / PG pairwise treatments on cell cycle phase distribution, as observed by 5-ethynyl-2′-deoxyuridine (EdU) incorporation and flow cytometry. While treatment with sertraline (7 μM) or gefitinib (10 μM) decreased active DNA synthesis in our GC cultures, pterostilbene (20 μM) increased EdU incorporation without a corresponding increase in cell number (Figure 5A, *p* < 0.05 and Supplementary Figure S5). Consistent with this, FACS analysis of 7-AAD stained and pterostilbene treated GCs showed a shift in DNA content with an increase in populations corresponding to S-phase and G2/M phase cells. The PS and PG combinations exerted a similar effect on the cell cycle phase distribution as pterostilbene alone (Figure 5B).

To study this effect in a larger number of cell lines, we analyzed automated microscopy data from 14 drug-treated GCs (Methods). From the images, we applied image analysis methods to obtain the distribution of DNA content in cells as detected by the Hoechst dye (Figure 5C). We subsequently applied linear regressions with G1, S and G2/M fractions as the response variables and the logarithm of the pterostilbene dose as the predictor variable. Of the 14 screened cell cultures, 12 had a negative slope for G1 phase (i.e. G1 phase cells decreased in response to pterostilbene) and a positive slope for S phase (sign test *p* = 9.16e-04) and 10 had a positive slope for G2/M phase (sign test *p* = 0.0283). In an extended analysis, we used linear mixed effects modeling to detect dose dependent changes in G1, S and G2/M populations that were common to all the cell cultures (Methods). The analysis confirmed a general decrease in G1 phase cells and a corresponding increase in S phase and G2/M phase cells (*p*-values 0.00014 for G1 phase, 1.17 × 10^−05^ for S phase, and 0.018 for G2/M phase). We thus conclude that pterostilbene treated GC cultures show clear signs of cell cycle arrest in the GC cells at 72 hours. Further investigation is needed to define the involved checkpoints.

**Figure 6:**
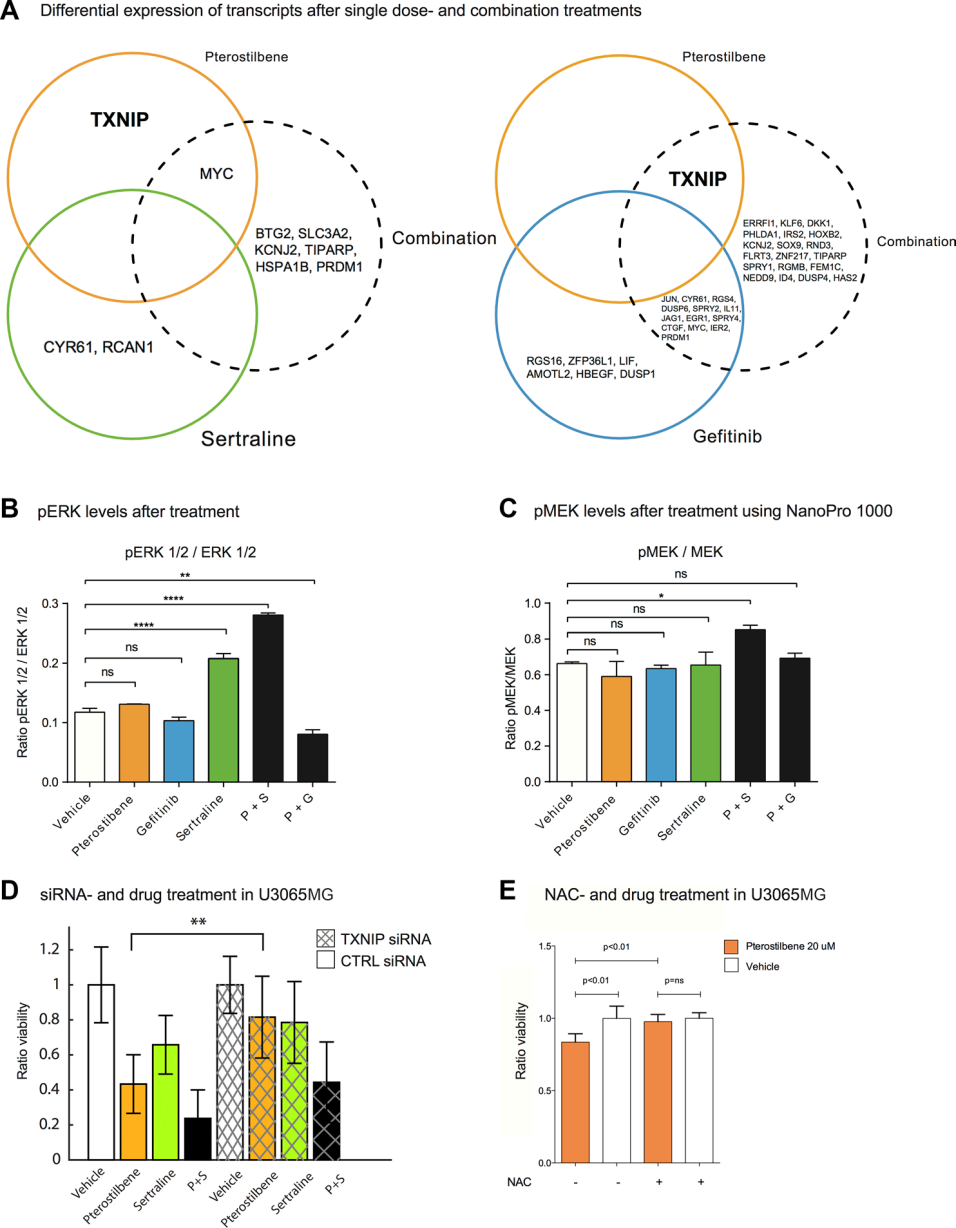
Transcriptional profiling after treatment reveals differentially expressed genes in response to PS and PG combinations We applied mRNA profiling to measure the response to single drugs and drug pairs after 1 hour of exposure in U3065MG. (**A**) Transcripts with significant changes for pterostilbene, sertraline and PS (left), and for pterostilbene, gefitinib and PG (right). The circles represent one treatment each and the overlap represents transcripts that are altered in both treatments. The transcripts displayed in the figure represent transcripts with a fold change of at least 0.2 and a significant fold change (adjusted *p*-value < 0.05) in U3065MG. All transcripts including fold changes and *p*-values are shown in Supplementary Table S3. Most transcripts are downregulated: all except TXNIP for pterostilbene in the pterostilbene areas and 4/7 (BTG2, SLC3A2, RCAN and HSPA1B) of the ones in the PS area (left). (**B**) pERK levels after 6 hour treatment using the NanoPro 1000 assay. pERK is significantly decreased after treatment with the PG combination, and significantly increased after treatment with the PS combination. (**C**) pMEK levels after 6 hour treatment. The PS combination significantly increased the pMEK levels. *p < 0.05, **p < 0.01, ***p < 0.001 (Student's *t*-test). (**D**) Modulation of pterostilbene effect during simultaneous knock down of TXNIP (24 and 48h experiments collected) (*p*-value obtained from linear model, Methods). (**E**) Pterostilbene effect after simultaneous treatment with NAC. *p < 0.05, **p < 0.01, ***p < 0.001 (Mann Whitney).

As an additional analysis of a possible mechanism of action, we applied whole transcriptome mRNA profiling to one GC culture (U3065MG after one hour of treatment). The transcriptional response was more pronounced (a higher number of differentially expressed transcripts) following treatment of PG compared to PS (Figure 6A and Supplementary Table S3). Among the differentially expressed genes in the PG treated cells (fold change > 0.2 and corrected *p* < 0.05) were MAPK negative feedback loop regulators DUSPs and SPRYs [35], suggesting interference with MAPK pathway by PG. Consistent with the transcriptional suppression of ERK targets (e.g. DUSP and SPRY family genes) in the transcriptional profiling experiment (Figure 6A), pERK levels, as measured by capillary electrophoresis, were reduced in GCs 6 hours after treatment by the PG combination (*p* < 0.01) (Figure 6B and signals in Supplementary Figure S6). Interestingly, the PS combination showed the opposite effect, inducing an increase of both pMEK and pERK (*p* < 0.05) (Figure 6B, 6C and signals in Figure S6). In addition to a likely perturbation of transcripts downstream of MAPK signaling, we noted that the mRNA for Thioredoxin interacting protein (TXNIP) was consistently upregulated after pterostilbene treatment. Given previous evidence of TXNIP as a tumor suppressor and mediator of ROS responses [36], we tested the hypothesis that the effect of pterostilbene might be dependent on TXNIP activation and/or induction of reactive oxygen species (ROS). To explore this idea, we tested if siRNA knockdown of TXNIP, or addition of the antioxidant N-acetylcysteine (NAC) could abrogate the effect of pterostilbene. Indeed, we found that downregulation of TXNIP counteracted the effect of pterostilbene (Figure 6D and Supplementary Figure S6C) as did NAC treatment (Figure 6E and Supplementary Figure S6C).

Taken together, the results highlight three components of the pterostilbene response in the GC culture U3065MG. First, pterostilbene applied singly and in combination with the two other compounds, induces cell cycle arrest in the GCs. Secondly, transcriptional and phosphoprotein evidence indicate that the expected effect of gefitinib on MAPK signaling is further elevated by pterostilbene. Thirdly, induction of TXNIP and an associated increase in ROS is a likely component of the response to pterostilbene treatment. Further exploration will be warranted to explore these findings *in vitro* and *in vivo*.

## DISCUSSION

We have established that pterostilbene can potentiate the anticancer effects in GCs of two drugs, the EGFR inhibitor gefitinib and the SSRI sertraline. Pterostilbene is regarded as a nontoxic compound and has good brain bioavailability [9–13]. Both EGFR inhibition and repurposing of sertraline have been proposed for GBM therapy, the latter as a component of a 9-drug combination protocol [23]. Our results warrant consideration of pterostilbene to be added to combinatorial treatment approaches involving sertraline. The association of PG to EGFR mutation and the CL subtype (which is characterized by EGFR amplification [25, 37]) are logical given that gefitinib targets EGFR, and we suggest pterostilbene potentiates the effect of gefitinib in a subset of Classical GBM. The findings suggest that pterostilbene may be a relevant potentiator of EGFR-inhibitors with good CNS penetration now under development.

Extending beyond previous studies, we show that PS and PG combinations suppress several GBM relevant phenotypes in GCs. While the exact mechanism remains to be determined, our results identify pterostilbene-driven cell cycle arrest, suppression of MAPK signaling and induction of TXNIP as points of action. These molecular changes translate to suppressed migratory and clone forming phenotypes, which are required for GBM progression [38]. The increase in pMEK and pERK following PS treatment is intriguing and may indicate a compensatory mechanism by which U3065MG up-regulate the MAPK pathway. This suggested that combined targeting by both PS and the MAPK pathway, for instance by an additional MEK inhibitor, might further enhance synergistic action. TXNIP, which was induced by pterostilbene, encodes a postulated tumor suppressor gene which plays an important role in oxidative homeostasis [36] and glioblastoma cell viability [39]. Previous studies have shown that pterostilbene treatment reduce the production ROS and attenuate glutamate-induced oxidative stress by enhancing the activities of the cellular anti-oxidants glutathione (GSH) and superoxide dismutase (SOD) through induction of translocation of the nuclear transcription factor erythroid 2 (NF-E2)-related factor 2 (Nrf2) [12]. TXNIP, which is controlled by Nrf2, on the other hand has been shown to promote ROS and apoptosis induction by inhibiting thioredoxin [40]. Upregulation of TXNIP in pterostilbene treated GCs therefore indicates a more complex response in tumor cells perhaps due to altered levels of oxidative stress or an altered metabolic state in glioma. This finding is supported by the protective effect of the antioxidant NAC on GC viability following pterostilbene treatment. The association between the ubiquitin-editing protein complex factor RNF11 and PS potentiation raise the hypothesis that the treatment may lead to Nrf2 ubiquitination and proteasomal degradation, allowing accumulation of TXNIP and ROS, which would in turn activate PI3K and PKC signaling pathways as well as ERK phosphorylation. In addition to these findings our transcriptional analysis of PS and PG treated cells identified other responding genes that may warrant follow-up work, including KCNJ2, which has previously been associated with modulation of both cell growth and drug resistance [41] and the MYC oncogene, which is a well-known transcriptional target of MAPK signaling which is previously reported to be over-expressed in gliomas [42, 43]. To fully deconvolute the mechanism behind the synergism of PS and PG will require a broader study *in vitro* and *in vivo*, across a broader set of doses, time-points and cell models, reserved for future work.

The significant correlation of CI with age for the PS combination (the CI is lower in young patients) could reflect inherent differences in the biology of GBMs in patients of different age [29]. It may also warrant closer investigation of methylation patterns of the cell cultures, given that the glioma CpG island methylator phenotype is more frequent in younger patients. However, this phenotype is also associated with IDH1 mutation, which was not observed among our cell cultures. The predictive potential of the RNF11 gene was characterized by a positive correlation with CI for the PS combination. This combination could thus be interesting to test in breast- and prostate cancer, due to the previously published association between RNF11 expression and these tumor types [32].

As far as we know, the association of the stratification of GBM patients for drug synergism has not been attempted before in a sizeable set of GC cultures, and our finding that marker transcripts and mutations in EGFR and PIK3CA correlate with drug pair synergism in GBM can have important applications for the design of preclinical and clinical studies. The successful prediction of PS synergism in a strict cross-validation analysis motivates prospective testing of such PS response in additional cases. From a methodology standpoint, our novel technique to determine CI from serial dilution of a drug pair in a fixed ratio is well suited for large-scale experimentation yet constitutes an approximation in the sense that it will be applicable to smooth response patterns with continuous changes in drug-drug interaction. The CI captures a large part of the interaction landscape, as compared to the interaction score measurements, pointing to the importance of evaluating more than one model when exploring drug interactions.

While a high number of GC cultures support that the synergism is a general phenomenon in GBM cells, it remains to be determined if the results hold *in vivo*; we reserve this for future work. In rodent models, pterostilbene has shown activity against hepatocellular, pancreatic and colorectal carcinomas [44–46], as well as melanoma [47]. Resveratrol (an analog of pterostilbene with lower bioavailability [9]) potentiated temozolomide in a heterotopic (flank) xenograft model of glioma [48]. This should motivate further investigation of pterostilbene and the PS and PG combinations in orthotopic xenograft models, using well-characterized glioma cells. Pterostilbene has been clinically evaluated for safety in human subjects [49], but remains to be systematically assessed as an anticancer agent in humans. It is also important to consider possible side effects of antioxidants, including stilbenoids, on tumor progression. For instance, experimental mice fed antioxidants have been reported to carry an increased tumor burden and increased rates of metastasis [50, 51]. A similar effect in glioma has not been reported, however one study indicates no consistent or significant association between antioxidant consumption and overall survival in glioblastoma patients [52]. Future work will be needed to evaluate the promise of pterostilbene-induced drug synergism *in vivo*.

## MATERIALS AND METHODS

### Glioblastoma cell cultures

The human malignant glioblastoma cell (GC) cultures are part of the Uppsala University Human Glioma Cell Culture (HGCC) bio bank [29]. GCs U3017MG, U3037MG, U3047MG and U3065MG were used for phenotypic characterization. An additional set of GCs were used to determine interaction scores (Supplementary Table S1 and [29]). Cells were cultured as previously described [7] and incubated at 37°C with 5% CO_2_.

### Compounds

Gefitinib (Tocris Biosciences), pterostilbene and sertraline (Enzo Biosciences) were dissolved in DMSO to a stock solution of 10mM and later diluted in cell medium to desired concentrations.

### Definition of interaction scores and combination index

We scored functional interaction between drugs by use of two scores, the interaction score (IS) and the Combination index (CI). IS, based on the Bliss independence model [53], was calculated as previously described [54]. IS is defined as = *w_ab_* − *w_a_ w_b_*, where *w_ab_* is the viability (relative to negative control) of cells following treatment by compounds a and b, and *w_a_*, *w_b_* are the corresponding effects of the single compounds. An observed value of IS significantly less than zero is consistent with potentiation. The combination index (CI), described by Chou and Talalay [55], in turn, is calculated from a given effect level y. CI_y_ is defined as X_a_/x_a_+X_b_/x_b_ where X_a_ and X_b_ are the doses of each drug constituting the combination that gives viability inhibition effect y, and x_a_ and x_b_ are the doses of each drug that individually achieves the effect y. CI < 1 is consistent with synergy, or potentiation. In the screening data, we calculated the average IS across all doses and the average CI across effect levels between 40% to 60% viability (Figure 3B, 3C). The two metrics are complementary indicators of functional interaction.

### Viability and cell growth assays

For Figure 1, cells were seeded at 12 000 cells/well in 96-well plates (BD Primaria) 24 hours prior to treatment. Cells were treated in triplicate at 3 different doses/drug as single doses and all doses in combination, resulting in 9 drug pair- and 6 single drug data points per combination studied (Figure 1). We measured viability by the resazurin-based assay Alamar blue (Life Technologies) and acquired imaged at 20X on an EVOS microscope (AMG). To monitor cell growth, U3065MG cells were seeded at 5000 cells/well in 96-well plates 24 hours prior to treatment with vehicle, single drugs and combinations. After starting treatment, cells were incubated in the IncuCyte instrument (Essen BioScience), and real-time density was recorded twice a day for 60 hours after which results were analyzed and presented as growth curves.

### Migration capacity

To evaluate migration capacity, 24-well plates with trans-well inserts were used (CBA-101-5, Cell Biolabs, Inc.), and coated with laminin for 30 min in 37°C before start of experiment. Cells were seeded at 300 000 cells/well together with treatments or DMSO controls. Experimental procedure and readout were performed as stated by manufacturer (images obtained at 20X on an EVOS microscope (AMG)).

### Gliomasphere forming assay

Cells were seeded at 2000 cells/well in BD 384 well plate (#353962) in suspension 24 hours prior to drug treatment. The cells were treated for 7 days and spheres were counted (Olympus bright field microscope) based upon their smooth shape and diameter using CellProfiler 2.1.0 and CPAnalyst [56, 57].

### Screening assay and high content imaging of 41 GC lines

Cells were seeded in 384-well microplates (BD Falcon Optilux #353962) 24 hours prior to treatment. Drugs and drug pairs were tested in 11-point dose dilution series (see Supplementary Table S2 and Supplementary Information), and assayed for viability after 72 hours of treatment using resazurin (R7017, SigmaAldrich) [58]. All data was normalized against DMSO vehicle wells. The effect on viability of each drug dose was calculated as a viability ratio W = Y_treated_/Y_control_, where Y represents the average fluorescence signal. Response curves were fitted using the package *drc* [59] in R [60]. The IS was calculated using doses represented in Supplementary Table S2, and the CI was calculated from effect levels 40–60% inhibition. Sigmoid response curves were fitted to the average viability ratio across all treated GCs, and the two single dose curves were used to calculate the predicted combination response (assuming no synergy). The observed and predicted combination response curves were then compared (statistics below).

To acquire images of the cell lines in the screen, plates were washed by PBS immediately after the resazurin assay, fixed by 4% PFA, and stained with the DNA-selective Hoechst 33342 stain (working solution 2 uM, #14533 Sigma-Aldrich). Plates were imaged on a Perkin Elmer Operetta instrument at 20x resolution (4 pictures per well). A set of 14 GC cultures (U3002MG, U3004MG, U3009MG, U3020MG, U3028MG, U3029MG, U3033MG, U3035MG, U3039MG, U3047MG, U3084MG, U3085MG, U3086MG and U3117MG) were analyzed by the CellProfiler software. Individual nuclei were identified and outlined based on intensity thresholding the Hoechst signal using the Otsu method. Touching or overlapping nuclei were split using the watershed method applied to the Hoechst intensity. To estimate the cell cycle subpopulations we used integrated DNA content, i.e. sum of intensities of the Hoechst stain inside each nucleus for each individual cell. Intensity data was subsequently binned to 100 intensity levels for each dose and manually gated to define G1, S and G2/M populations (c.f. Figure 5). To statistically test for dose-dependent changes in cell cycle phase for the whole sample of 14 cell cultures, we employed a linear mixed effects model, in which the fraction of cells in phase G1 was modeled by: *y*_*i*_^*G*1^ = *c^FIXED^* + *β^FIXED^ x* + *c_j_^RANDOM^* + *β_j_^RANDOMX^*. The ‘FIXED’ terms capture common effects for all cell cultures, and the ‘RANDOM’ effects capture additional effects that are specific to each cell culture. Fitting the model (Matlab FITLME) we obtained an estimate, a confidence interval and a *p* value for the common slope effect (Figure 5C bottom right panel). As a complement we also explored separate linear regressions for each cell line, and the distribution of slopes *β^FIXED^* (Figure 5C, middle figure).

### Prediction of combination index from RNA profiles, DNA copy number aberration profiles and somatic mutations

We obtained normalized gene-level log2 relative transcript profiles (Affymetrix HTA 2) and gene-level log2 relative DNA copy number aberration profiles (Affymetrix Cytoscan HD) from the U3000/HGCC consortium at Uppsala University (hgcc.se). Using each respective data set as a covariate (x) and the CI as the response variable (y) we used elastic net regression to fit a linear function $y_{i}=f\left(x_{i}\right)=\beta_{0}+\sum_{j=1}^{p}\beta_{j}X_{ij}$ where i is the index of the patient, *β*_0_ is a constant, *β* = {*β*_1_, *β*_2_, …, *β*_p_} is the p-dimensional parameter weight vector and p is the number of genes. For the N patients, the elastic net function optimizes, $\frac{1}{2N}\overset{N}{\underset{I=1}{\sum }}{\left(y_{i}-f(X_{i})\right)}^{2}+\lambda \overset{p}{\underset{j=1}{\sum }}(\frac{1-\alpha }{2}\beta_{i}^{2}+\alpha |\beta_{j}|)$ where lambda and alpha are regularization parameters (lambda determines the degree of regularization and alpha determines the balances between a least-squares (alpha = 0) and a lasso (alpha = 1) penalty. We used a default value of alpha = .9 and tuned lambda by leave-one-out cross validation (selecting the lambda with the highest Pearson correlation between left-out observed and left-out predicted values. The genes shown in Figure 4 are the genes that were most frequently selected as predictive variables (*β_j_* ≠ 0 for the corresponding gene j) across the N different leave-out simulations. The correlations and scatter plots shown in Figure 4 represent leave-one-out results at the optimal lambda. To correlate with mutation status, we used preliminary calls from the U3000/HGCC pipeline that represent AnnoVar-assigned consensus calls applied to Ion Torrent whole exome data (cell line DNA with patient matched reference blood), calling mutations as positive if detected by 3 or 4 mutation callers from a panel consisting of the four callers (MuTect, VarScan2, Somatic Sniper, Torrent Variant Caller). Only mutations predicted by AnnoVar to be missense were included in the analysis.

### EdU incorporation assay

We applied an EdU-based assay (Invitrogen, Molecular probes, c10337) to assess proliferation. GCs were seeded at 10000 cells/well on laminin-coated 96 well plates (Greiner bio-one, #655986) 24 hours prior to treatment. Readout was performed at 24- and 48 hours using an 8-hour EdU exposure according to manufacturer's protocol. Images were acquired using the ImageXpress (20X) (Molecular devices) and were counted for total number of cells (Hoechst 33342) and the percentage of proliferating cells (EdU incorporated) using CellProfiler2.1.0 and CPAnalyst [56, 57].

### Cell cycle analysis

Cells were seeded at 400 000 cells/well in 6-well plates (BD Primaria, BD Biosciences) and incubated 24 hours before start of treatment. The cells were treated for 24 hours, washed in PBS, detached and fixed in ice-cold ethanol. Fixed cells were washed with PBS and re-suspended in 400 μl of Vindelov's reagent (20 mM Tris-HCl pH 8, 100 mM NaCl, 1 μg/mL 7-AAD, 20 μg/mL RNase, 0.1% NP40) and incubated for 30 minutes at 37°C. The cells were then run and analyzed (phases manually set from control cells) on a BD Accuri*™* C6 Flow Cytometer (BD Biosciences).

### RNA sequencing of treated cells

GCs were seeded at 500 000 cells/well in 6-well BD Primaria plates 24 hours prior to treatment. Cells were treated for 1 hour before harvest. RNA for all experiments was extracted and purified according to protocol (RNeasy Plus Mini-kit, Qiagen). A total of 3 μg RNA was used in the preparation of the TruSeq library, for which an Illumina Low-Throughput TruSeq RNA Sample Preparation Kit protocol was used. Samples were sequenced on an Illumina HiSeq 2000 sequencer as single-end 51-nucleotide reads according to the manufacturer's protocol. Data was handled using STRT software [61]. The R software was used to define groups of responding transcripts (Figure 6A and Supplementary Table S3).

### Protein expression assay

NanoPro 1000 analysis was used to assess ERK 1/2 and MEK 1/2 activity. The protocol was modified from [62]. Samples were loaded into the NanoPro 1000 System (ProteinSimple, Santa Clara, USA) and probed with anti-ERK 1/2, anti-pERK 1/2, pMEK and MEK 1/2. Full procedure in Supplementary Information.

### Gene knock down experiments and NAC treatments

U3065MG and U3027MG were used for knock down studies, and U3065MG for the NAC experiment. RNF11 (Cat#AM16708, Art nr. 134278) and TXNIP (Cat#AM16708, Art nr. 1358498) siRNAs were purchased from Thermo Scientific/Life Technologies. For knock down validation, primers were purchased from Thermo Scientific/Life Technologies (Hs02801538_g1 for RNF11 and Hs01006900_g1 for TXNIP). N-acetylcysteine (Art nr. A9165) was purchased from Sigma Aldrich. For knock down experiments, cells were seeded in 24-well plates 24 hours prior to addition of siRNA. At 24 and 48 h hours after siRNA addition, drugs and drug pairs were added and viability read out was performed 48 hours after treatment. For the NAC experiment, cells were seeded at 5000 cells/well in 96-well plates 24 hours prior to pre-treatment with NAC (10 mM). After one hour of NAC treatment, pterostilbene was added to the media and viability readout was performed 48 hours after treatment start. Experiments were performed in 5 replicates and statistically assessed using a linear model in which the viability response was modeled as *w* = *w*_0_ + Δ*w_siRNA_* + Δ*w_drug_* + Δ*w_siRNA, drug_*, where *w*_0_ is the baseline viability, Δ*w_siRNA_* is the viability changed induced by the siRNA, Δ*w_drug_* is the viability change induced by the drug, and Δ*w_siRNA, drug_* is the viability change caused by siRNA and drug interaction, respectively. *P*-values and confidence intervals (Figure 4D and Figure 6D, 6E) were obtained from the fitted model (Matlab glmfit).

### Statistical assessment of phenotypic responses

Normalized response ratios for all assays were calculated as Y_treated_/Y_control_, where Y represents the raw response values. To assess statistical significance of the interaction scores in Figure 1, we performed a permutation test by randomizing the treatment labels 10 000 times, thus obtaining a distribution of the interaction scores simulating the case of no functional interaction. From this distribution we obtain the empirical *p*-value. To assess the significance of a functional interaction score in the treated 41 GCs, the non-parametric Wilcoxon signed rank test was used to determine if median IS and CI differed from 0 and 1, respectively. For phenotypic assays, differences between groups were assessed using the non-parametric Mann Whitney test in the GraphPad Prism software. The differences in expression in the NanoPro assay were assessed using a Student's Independent *t*-test.

### DNA sequencing data

We obtained whole exome sequencing data for genes EGFR, NF1, PDGFRA, PIK3CA, PIK3R1, PTEN, RB1, RYR2, TP53, TTN from the Human Glioma Cell Culture (hgcc.se consortium). In short, the data represents 100X Ion Torrent sequencing data of cell line DNA following Agilent SureSelect whole exome capture. Patient-matched blood was used as control. Aggregated mutation calls from MuTect, Somatic Sniper, VarScan and Torrent Suite were processed by Annovar to define somatic missense variants. only variants detected by 3 or 4 callers were retained. We excluded known SNPs (variants with an assigned rs ID, and/or found in the 1000 genomes project at frequencies above 1/200). For more information regarding these data we refer to hgcc.se.

## SUPPLEMENTARY MATERIALS TABLES FIGURES




