# Supplementary material for: Case-specific potentiation of glioblastoma drugs by pterostilbene
Source: Oncotarget. 2016 Sep 28;7(45):73200–15. doi: 10.18632/oncotarget.12298 (PMC5341973; doi:10.18632/oncotarget.12298)
Supplement: Supplementary file 1 [file oncotarget-07-73200-s001.pdf]

Case-specific potentiation of glioblastoma drugs by pterostilbene

Supplementary Materials

Glioblastoma cell cultures from the Uppsala HGCC biobank

Isolation, propagation, establishment of adherent glioblastoma cell (GC) cultures (all used in Supplementary Table S2), and characterization of stem cell markers and tumor initiating capacity of all GCs in the HGCC biobank is accounted for in [1]. The same reference also states the subtyping schedule for all GCs, based on the Verhaak subtyping gene sets described in [2].

Immortalized human astrocytes were a kind gift from the Arne Östman laboratory at Karolinska Institute.

Proliferation assessed by the real time cell analyzer (RTCA) xCELLigence

The xCELLigence Real-Time Cell Analyzer (RTCA) (Roche) was used to measure long-time proliferation. The xCELLigence cell index (CI) impedance real-time measurements were performed according to the manufacturer's protocol. GC U3037MG was seeded at 10 000 cells/well, in a 16-well E-plate VIEW16 (ACEA Biosciences) 24 hours before addition of pterostilbene (20 µM) and DMSO control. The real-time proliferation studies were performed with treatment during approximately 80 hours.

Extended screening procedure in 41 GCs

Cells were seeded in 384-well microplates (BD Falcon Optilux Cat. #353962) 24 hours prior to treatment using a Multidrop 384 liquid dispenser (ThermoScientific, Sweden). To ensure growth phase at end of the assay (~70% confluency) cells were seeded at a density ranging between 2000 – 4000 cells/well. Single drugs and drug pairs were transferred using the ECHO550 non-contact liquid dispenser (Labcyte, USA) to a 384 V-bottom polypropylene plate. The drugs were diluted in medium and transferred using the MDT 384 head on a Janus automated workstation (PerkinElmer, USA) to the cell plates. Gefitinib, sertraline, pterostilbene, PS (10 mM at 1:0.35) and PG (10 mM at 1:0.5) were tested in 11-point dose dilution series (see Supplementary Table S2), and assayed for viability after 72 hours of treatment on an EnVision Multilabel reader (PerkinElmer, USA) using resazurin (R7017, SigmaAldrich) (Nociari). As a positive control, the drug doxorubicin was screened with the same dose response curve setting. All data was normalized against plate DMSO control wells. The effect on viability of each drug dose was calculated as a viability ratio $W = Y_{treated} / Y_{control}$, where Y represents the average fluorescence signal. A four parameter sigmoid model (4-PL) was fitted for each dose response series using the package drc in R. The four parameter (p1, p2, p3 and p4) dose response model is defined as $f(X)=\frac{1+P^{2}-p^{1}}{1+e^{p3(X-P4)}}$, where x is the dose and f(x) is the response.

Protein expression analysis using NanoPro 1000

Cells were lysed in Bicine/CHAPS buffer containing phosphatase and protease inhibitors (ProteinSimple, Santa Clara, CA). Protein concentration was measured by using BCA Protein Assay Kit (Pierce, Rockford, IL, USA). Samples were run in duplicates. Lysates were mixed with ampholyte premix (040–972, G2 pH 5–8) and fluorescent pI standards (040–646, pI Standard Ladder 3) before being loaded into the NanoPro 1000 system (ProteinSimple, Santa Clara, CA) for analysis. Isoelectric focusing was performed in capillaries filled with a mixture of cell lysate (0.1 – 0.15 µg/µl protein), fluorescently labeled pI standards, and ampholytes. The separated proteins were cross linked into the capillary wall by using UV light followed by immunoprobing with anti-ERK 1/2 (Cell Signaling Technology, # 9102, 1:100) anti-pERK 1/2 (Cell Signaling Technology, # 9101, 1:50), pMEK (Abcam, #ab32088, 1:40) and MEK 1/2 (Santa Cruz Biotechnology, #sc-436, 1:100). HRP-conjugated secondary antibodies were used from Jacksson ImmunoResearch (Donkey Anti-Rabbit IgG, # 711-035-152) to detect the signal. The signal was visualized by ECL and captured by a CCD camera. The digital image was analyzed and peak area quantified with Compass software (ProteinSimple, Santa Clara, CA).

REFERENCES

1. Xie Y, et al. The Human Glioblastoma Cell Culture Resource: Validated Cell Models Representing All Molecular Subtypes. EBioMedicine. 2015; 2:1351–63.
2. Verhaak RG, et al. Integrated genomic analysis identifies clinically relevant subtypes of glioblastoma characterized by abnormalities in PDGFRA, IDH1, EGFR, and NF1. Cancer Cell. 2010; 17:98–110.

A **Structure formula for the three drugs studied**

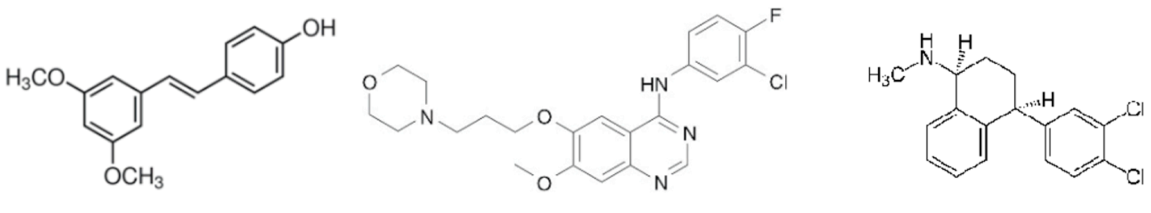

Pterostilbene **Gefitinib** **Sertraline**

B **Gliomasphere formation, all tested doses**

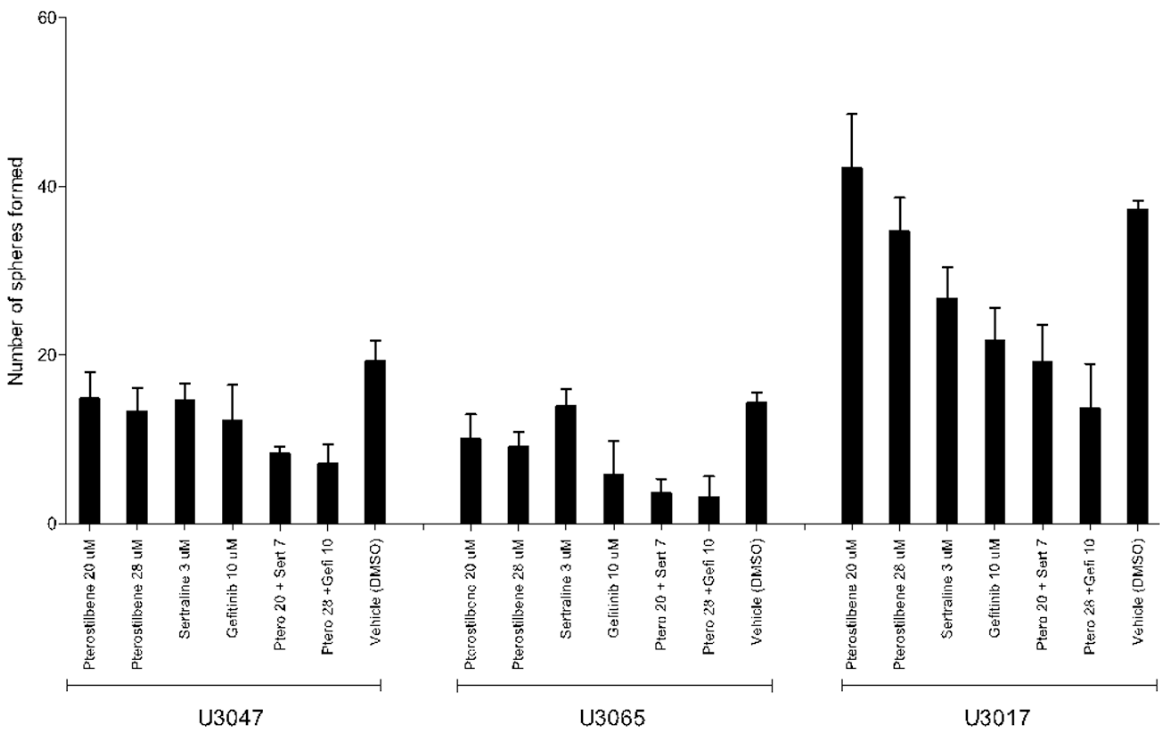

Supplementary Figure S1: (A) Chemical structure of used compounds, pterostilbene, sertraline and gefitinib. (**B**) Gliomasphere formation with all doses tested. Graphs presented as mean and SD.

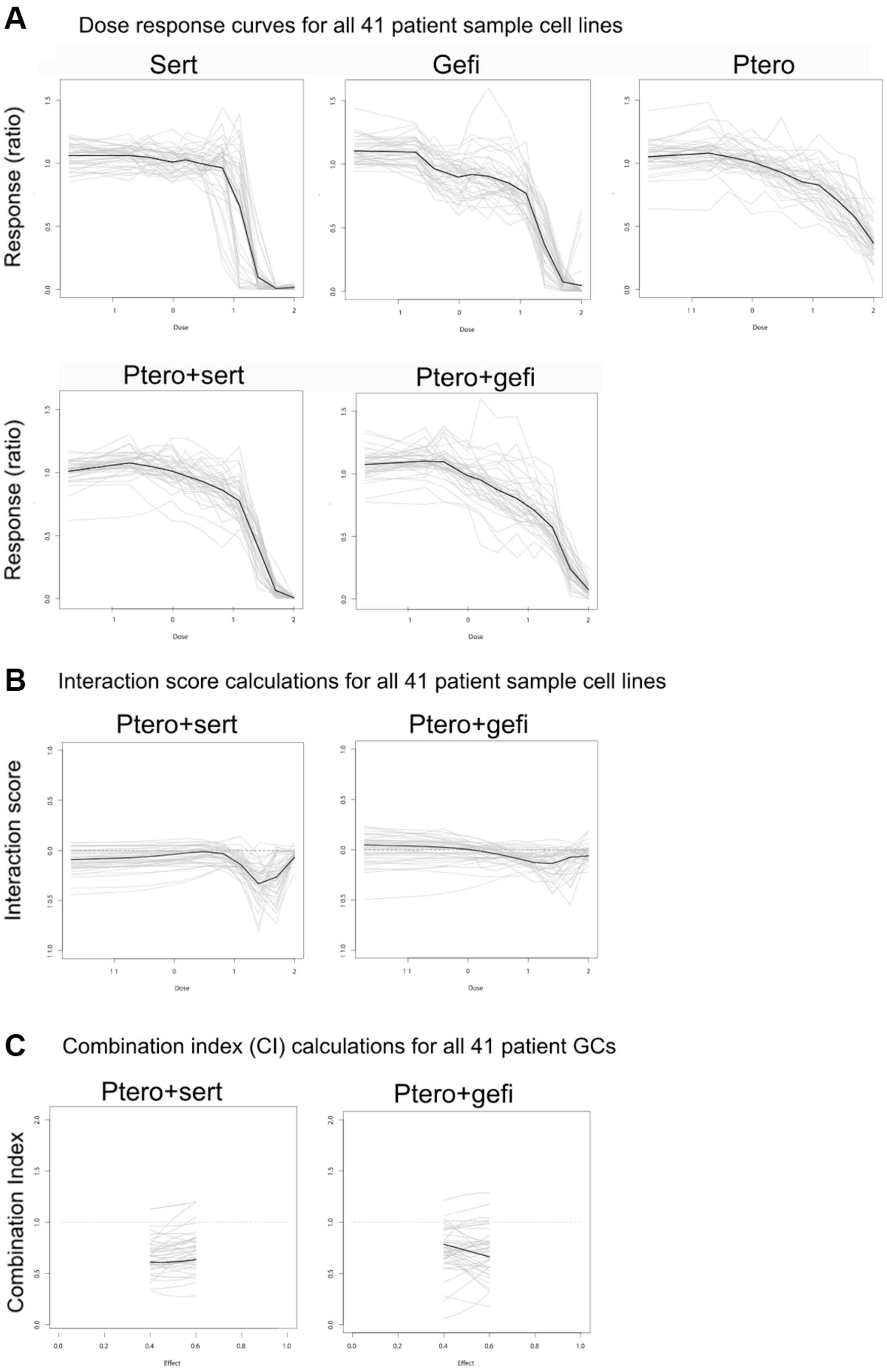

Supplementary Figure S2: (**A**) Dose response profiles for all single drugs for each individual patient-derived GC culture**.** Grey lines indicate each individual response and black line indicate average over all 41 patient-derived GCs. (**B**) Interaction scores for the two combinations for each individual patient cell line (grey lines) and the interaction scores for the average dose response curve over all patients (black line). (**C**) Same as (B), but using combination index at effects 40–60%.

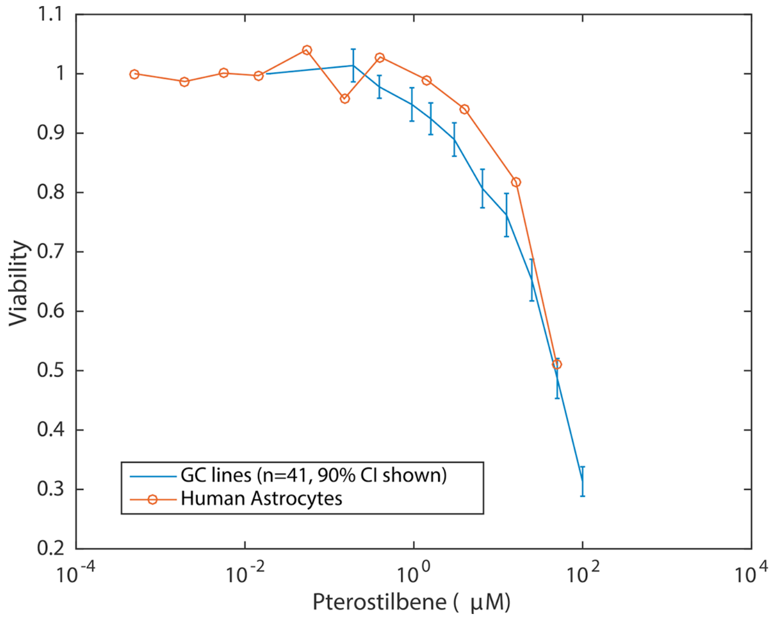

Supplementary Figure S3: Pterostilbene effect on viability across 41 glioblastoma cells (GC lines) and human astrocytes. Normalized viability on y-axis and dose on x-axis (µM). Bars indicate a 95% confidence interval of the mean viability for the GC lines

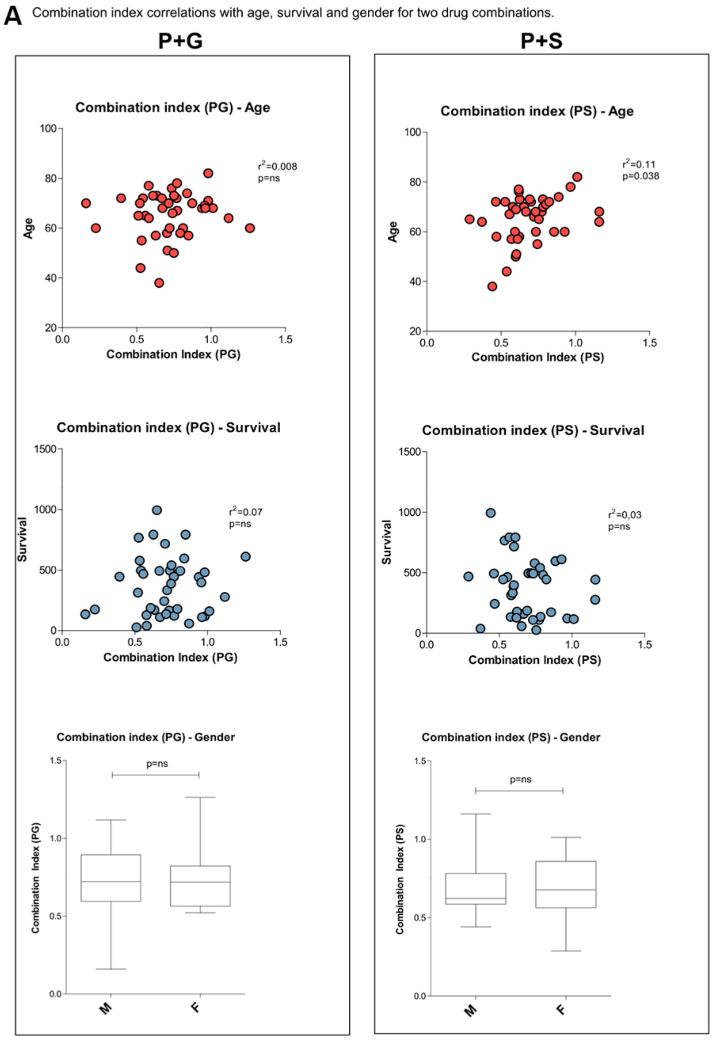

Supplementary Figure S4 : A Correlation analysis using Combination Index (CI) for both PS (right) and PG (left). CI correlated with age (top), survival (middle) and gender (bottom). The r2 and *p*-value are displayed in the graphs. Statistics was done using GraphPad Prism (Pearson's for age and survival and Student's Independent *t*-test for gender).

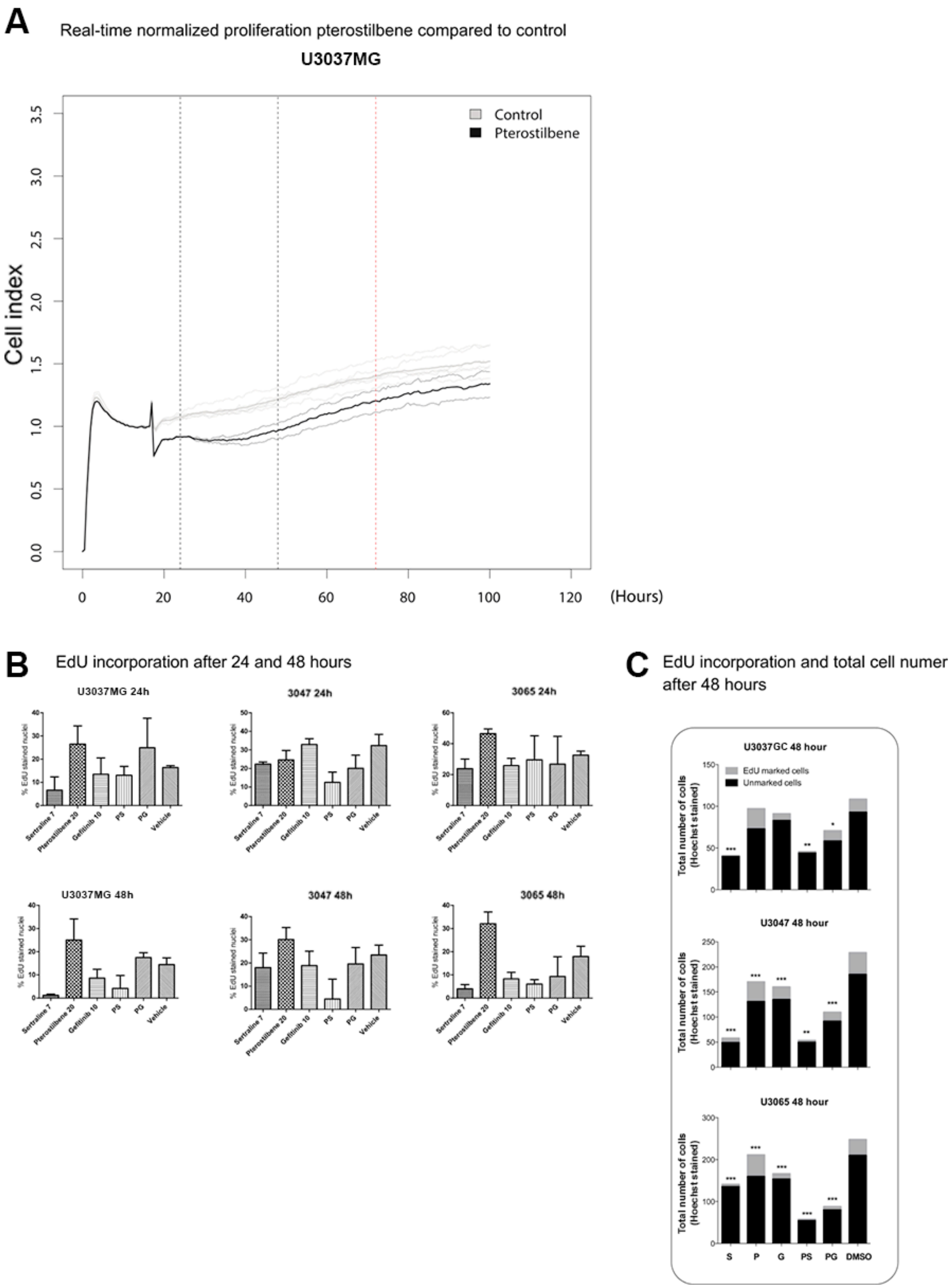

Supplementary Figure S5 : (**A**) Cell index measurements (corresponding to number of cells in one well) was recorded for approximately 100 hours (approximately 80 hours with drug)**.** The Cell index was compared over time, at 30 minutes intervals, between vehicle (DMSO) and 20 µM pterostilbene to assess real-time proliferation. Each dotted line is 24 hours, and the red indicates 72 hours. Drugs were added close to the 24-hour time point. Grey line indicates control (mean, SD) and black line indicates pterostilbene (mean, SD) for each time point. (**B**) Fraction of EdU marked cells in U3037MG, U3047MG and U3065MG after 24− and 48 hour treatment (graphs represent mean and 95% CI). (**C**) Total number of cells and fraction of EdU marked cells in U3037MG, U3047MG and U3065MG after 48 hour treatment. Data is presented as means (triplicate measurements) of number of Hoechst stained cells (black fraction) and number of Hoechst stained cells with an EdU stain (grey fraction). *$p < 0.05$, **$p < 0.01$, ***$p < 0.001$ using Student's Independent *t*-test.

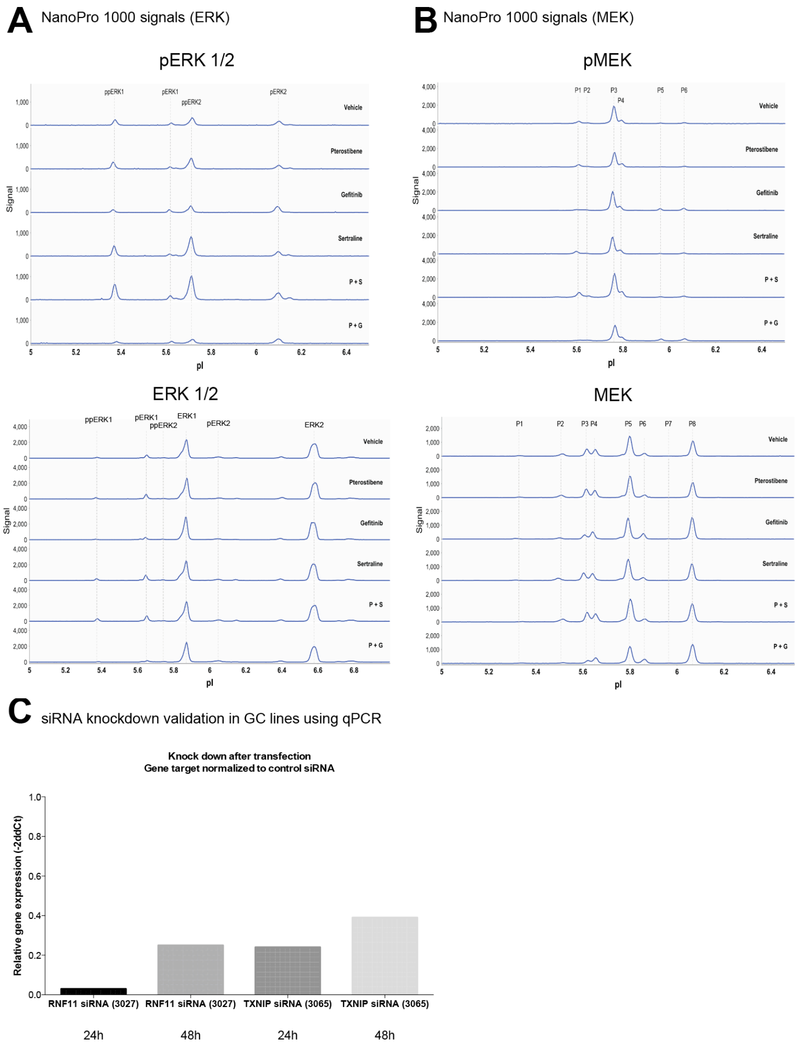

Supplementary Figure S6 : Signals from the NanoPro 1000 assay and p-protein results after pterostilbene treatment and validation of target gene reduction. Representative electropherogram showing pERK, ERK (**A**) and pMEK, MEK (**B**) protein peaks and (**C**) qPCR validations of siRNA knockdown of RNF11 and TXNIP in U3027 and U3065, respectively.

Supplementary Table S1: All glioblastoma cell cultures, their corresponding subtypes batch

GCs 1)	Subtype 2)
**U3013 1**	PN
**U3017 1**	CL
**U3024 1**	MS
**U3037 1**	MS
**U3065 1**	CL
**U3084 1**	CL
**U3002 2**	CL
**U3004 2**	NA
**U3005 2**	PN
**U3009 2**	CL
**U3020 2**	MS
**U3035 2**	MS
**U3008 3**	NL
**U3016 3**	PN
**U3021 3**	NL
**U3027 3**	MS
**U3031 3**	MS
**U3046 3**	MS
**U3029 4**	MS
**U3028 4**	CL
**U3033 4**	PN
**U3039 4**	CL
**U3047 4**	PN
**U3085 4**	NL
**U3019 5**	PN
**U3034 5**	MS
**U3056 5**	CL
**U3082 5**	PN
**U3086 5**	CL
**U3117 5**	PN
**U3053 6**	MS
**U3054 6**	MS
**U3060 6**	MS
**U3073 6**	MS
**U3078 6**	MS
**U3088 6**	MS
**U3051 7**	MS
**U3062 7**	MS
**U3068 7**	MS
**U3110 7**	MS
**U3118 7**	PN
**U3129 7**	NA

1) GC, batch number in screen.

2) Subtypes MS=mesenchumal, PN=proneural, CL=classical, NL=neural, NA=not available

Supplementary Table S2: Corresponding single doses for proportions of combination mix (uM)

		Doses (uM) for single drugs in screen at a stock of 10 mM.										
		0,019	0,192	0,39	0,95	1,59	3,02	6,51	12,54	25	50	100
**Conc. in stock (mM)**	**Drug**											
3,4	Gefitinib	0,01	0,07	0,13	0,32	0,54	1,03	2,21	4,26	8,50	17,00	34,00
2,6	Sertraline	0,00	0,05	0,10	0,25	0,41	0,79	1,69	3,26	6,50	13,00	26,00
6,6	Pterostilbene	0,01	0,13	0,26	0,63	1,05	1,99	4,30	8,28	16,50	33,00	66,00
7,4	Pterostilbene	0,01	0,14	0,29	0,70	1,18	2,23	4,82	9,28	18,50	37,00	74,00
**Combination mix drugs**	**Sertraline**	**Gefitinib**	**Pterostilbene**	**Total drug conc in mix.**	**Proportions**							
Ptero+sert (7,4 × 2,6 mM)	2,6 mM		7,4 mM	10 mM	1 : 0.35	1 : 0.35						
Ptero+gefi (6,6 × 3,4 mM)		3,4 mM	6,6 mM	10 mM		1 : 0.5						

Supplementary Table S3: Gene expression after treatment with single doses and combination in U3065MG. See Supplementary_Table_S3
