# Supplementary material for: Case-specific potentiation of glioblastoma drugs by pterostilbene
Source: Oncotarget. 2016 Sep 28;7(45):73200–15. doi: 10.18632/oncotarget.12298 (PMC5341973; doi:10.18632/oncotarget.12298)
Supplement: Supplementary file 2 [file oncotarget-07-73200-s002.docx]

**Table S3.** Gene expression after treatment with single doses and combination in U3065

**Pterostilbene + Gefitinib**

| **Gene symbol** | **Fold change** | **Fold change** | **Fold change** | **Adjusted p-value** | **Adjusted p-value** | **Adjusted p-value** |
| --- | --- | --- | --- | --- | --- | --- |
|  | **Gefitinib** | **Pterostilbene** | **P+G** | **Gefitinib** | **Pterostilbene** | **P+G** |
| ERRFI1 | -0,1586 | -0,0275 | -0,2816 | 0,1327 | NA | 0,0000 |
| JUN | -0,2480 | -0,0019 | -0,3653 | 0,0000 | 0,9892 | 0,0000 |
| CYR61 | -0,4711 | -0,1394 | -0,6664 | 0,0000 | NA | 0,0000 |
| TXNIP | 0,0305 | 0,2299 | 0,2945 | 1,0000 | 0,0000 | 0,0000 |
| RGS4 | -0,2247 | -0,0430 | -0,2320 | 0,0008 | NA | 0,0048 |
| RGS16 | -0,2643 | 0,0893 | -0,1061 | 0,0000 | NA | 0,9981 |
| KLF6 | -0,1021 | -0,0937 | -0,2357 | 1,0000 | NA | 0,0001 |
| DKK1 | -0,1484 | -0,0351 | -0,2798 | 0,0669 | 0,7922 | 0,0000 |
| PHLDA1 | -0,1758 | -0,0041 | -0,3147 | 0,0000 | 0,9743 | 0,0000 |
| DUSP6 | -0,4063 | -0,1042 | -0,6297 | 0,0000 | NA | 0,0000 |
| SPRY2 | -0,2778 | -0,0710 | -0,4109 | 0,0000 | 0,3986 | 0,0000 |
| IRS2 | -0,1244 | -0,0364 | -0,2338 | 0,8486 | NA | 0,0004 |
| ZFP36L1 | -0,2191 | 0,1295 | -0,1114 | 0,0003 | NA | 0,9807 |
| HOXB2 | -0,1731 | 0,0166 | -0,2166 | 0,0070 | 0,9168 | 0,0002 |
| KCNJ2 | -0,1987 | -0,0460 | -0,2919 | 0,0002 | 0,6810 | 0,0000 |
| SOX9 | -0,0626 | 0,0019 | -0,2565 | 1,0000 | 0,9892 | 0,0000 |
| IER2 | -0,2100 | 0,0146 | -0,1963 | 0,0035 | NA | 0,0432 |
| IL11 | -0,2970 | 0,0167 | -0,2669 | 0,0000 | NA | 0,0002 |
| RND3 | -0,0702 | -0,1132 | -0,2778 | 1,0000 | NA | 0,0001 |
| JAG1 | -0,2014 | 0,0541 | -0,2728 | 0,0003 | 0,6329 | 0,0000 |
| FLRT3 | -0,0583 | -0,0823 | -0,2498 | 1,0000 | NA | 0,0013 |
| ZNF217 | -0,0366 | -0,0336 | -0,2028 | 1,0000 | NA | 0,0380 |
| LIF | -0,2122 | -0,0192 | -0,2427 | 0,0009 | NA | NA |
| AMOTL2 | -0,2252 | 0,0195 | -0,1671 | 0,0003 | NA | 0,1187 |
| TIPARP | -0,0650 | -0,3044 | -0,2319 | 1,0000 | NA | 0,0031 |
| SPRY1 | -0,1622 | -0,0431 | -0,2846 | 0,1327 | NA | 0,0001 |
| RGMB | -0,0907 | 0,0116 | -0,2017 | 1,0000 | NA | 0,0048 |
| FEM1C | -0,1541 | -0,0589 | -0,2202 | 0,0905 | NA | 0,0005 |
| EGR1 | -1,0384 | -0,1442 | -1,0809 | 0,0000 | NA | 0,0000 |
| HBEGF | -0,2363 | -0,0432 | -0,2032 | 0,0001 | NA | NA |
| SPRY4 | -0,2461 | 0,0261 | -0,3386 | 0,0001 | NA | 0,0000 |
| DUSP1 | -0,2485 | -0,0036 | -0,1549 | 0,0000 | NA | NA |
| NEDD9 | -0,0707 | -0,0845 | -0,2287 | 1,0000 | NA | 0,0018 |
| ID4 | -0,1173 | -0,0163 | -0,2248 | 1,0000 | NA | 0,0018 |
| PRDM1 | -0,1982 | -0,0091 | -0,4427 | 0,0080 | NA | 0,0000 |
| CTGF | -0,2611 | -0,0594 | -0,2667 | 0,0000 | 0,6867 | 0,0001 |
| DUSP4 | -0,1132 | -0,0604 | -0,2359 | 1,0000 | 0,6164 | 0,0001 |
| HAS2 | -0,0161 | -0,0726 | -0,2323 | 1,0000 | NA | 0,0036 |
| MYC | -0,2579 | -0,0521 | -0,3132 | 0,0000 | NA | 0,0000 |

**Pterostilbene + Sertraline**

| **Gene symbol** | **Fold change** | **Fold change** | **Fold change** | **Adjusted p-value** | **Adjusted p-value** | **Adjusted p-value** |
| --- | --- | --- | --- | --- | --- | --- |
|  | **Pterostilbene** | **Sertraline** | **P+S** | **Pterostilbene** | **Sertraline** | **P+S** |
| **CYR61** | -0,1394 | -0,2819 | -0,1484 | NA | 0,0000 | 0,0751 |
| **TXNIP** | 0,2299 | -0,1014 | 0,1568 | 0,0000 | 0,9998 | 0,0977 |
| **BTG2** | 0,0607 | 0,0273 | 0,2112 | NA | 0,9998 | 0,0133 |
| **SLC3A2** | 0,1122 | 0,1452 | 0,2835 | NA | 0,9998 | 0,0000 |
| **KCNJ2** | -0,0460 | -0,0577 | -0,2204 | 0,6810 | 0,9998 | 0,0000 |
| **RCAN1** | -0,0391 | 0,2512 | 0,0589 | NA | 0,0000 | 0,9999 |
| **TIPARP** | -0,3044 | -0,0062 | -0,2553 | NA | 0,9998 | 0,0003 |
| **HSPA1B** | 0,0606 | 0,0754 | 0,2446 | NA | 0,9998 | 0,0005 |
| **PRDM1** | -0,0091 | -0,0957 | -0,3249 | NA | 0,9998 | 0,0000 |
| **MYC** | -0,0521 | -0,2047 | -0,2162 | NA | 0,0253 | 0,0092 |
